# CDG: An Online Server for Detecting Biologically Closest Disease-Causing Genes and its Application to Primary Immunodeficiency

**DOI:** 10.3389/fimmu.2018.01340

**Published:** 2018-06-27

**Authors:** David Requena, Patrick Maffucci, Benedetta Bigio, Lei Shang, Avinash Abhyankar, Bertrand Boisson, Peter D. Stenson, David N. Cooper, Charlotte Cunningham-Rundles, Jean-Laurent Casanova, Laurent Abel, Yuval Itan

**Affiliations:** ^1^St. Giles Laboratory of Human Genetics of Infectious Diseases (Rockefeller Branch), The Rockefeller University, New York, NY, United States; ^2^Graduate School, Icahn School of Medicine at Mount Sinai, New York, NY, United States; ^3^Department of Medicine, Division of Clinical Immunology, Icahn School of Medicine at Mount Sinai, New York, NY, United States; ^4^New York Genome Center, New York, NY, United States; ^5^Laboratory of Human Genetics of Infectious Diseases (Necker Branch), INSERM U1163, Paris, France; ^6^Paris Descartes University, Imagine Institute, Paris, France; ^7^Institute of Medical Genetics, School of Medicine, Cardiff University, Cardiff, United Kingdom; ^8^Howard Hughes Medical Institute, New York, NY, United States; ^9^Pediatric Immunology-Hematology Unit, Necker Hospital for Sick Children, Paris, France; ^10^The Charles Bronfman Institute for Personalized Medicine, Icahn School of Medicine at Mount Sinai, New York, NY, United States; ^11^Department of Genetics and Genomics, Icahn School of Medicine at Mount Sinai, New York, NY, United States

**Keywords:** disease-causing gene, gene filtering, next-generation sequencing, genomics, human gene connectome

## Abstract

High-throughput genomic technologies yield about 20,000 variants in the protein-coding exome of each individual. A commonly used approach to select candidate disease-causing variants is to test whether the associated gene has been previously reported to be disease-causing. In the absence of known disease-causing genes, it can be challenging to associate candidate genes with specific genetic diseases. To facilitate the discovery of novel gene-disease associations, we determined the putative biologically closest known genes and their associated diseases for 13,005 human genes not currently reported to be disease-associated. We used these data to construct the closest disease-causing genes (CDG) server, which can be used to infer the closest genes with an associated disease for a user-defined list of genes or diseases. We demonstrate the utility of the CDG server in five immunodeficiency patient exomes across different diseases and modes of inheritance, where CDG dramatically reduced the number of candidate genes to be evaluated. This resource will be a considerable asset for ascertaining the potential relevance of genetic variants found in patient exomes to specific diseases of interest. The CDG database and online server are freely available to non-commercial users at: http://lab.rockefeller.edu/casanova/CDG.

## Introduction

Genetic mutations have been found to underlie a large number of inherited human diseases. In the past decade, refinements in next-generation sequencing techniques (NGS) have made it possible to detect the full set of gene variants in patients. The average human genome contains about 20,000 coding variants and hundreds of thousands of non-coding variants (1). A common approach to identify candidate variants for further investigation from NGS data involves screening for those in known disease-causing genes (2–4). However, variants in novel disease-associated genes should be estimated by computational predictions (5).

Databases such as the Human Gene Mutation Database [HGMD (6)], and ClinVar (7, 8) provide manually curated information about mutations in known disease-causing genes, also known as the Clinome (9). Several methods including the Search Tool for the Retrieval of Interacting Genes/Proteins [STRING (10)], Exomiser that prioritizes genetic variants from a vcf file (11), the Probabilistic functional gene network of Homo Sapiens [HumanNet (12)], and Functional Coupling [FunCoup (13, 14)] can be used to assess human genes directly connected to candidate genes. The human gene connectome [HGC (15)] extends these approaches by prioritizing candidate genes according to their computed biological distances from known disease-causing genes.

We generated a complementary resource, the closest disease-causing genes (CDG) database and server to identify novel gene-disease associations. CDG computes the biologically closest known disease-causing genes and corresponding diseases for 13,005 human candidate genes not currently observed to be disease-causing, allowing investigators to associate these candidate genes with known disease phenotypes. We demonstrate the efficiency of this method in five patients with various primary immunodeficiencies and modes of inheritance, significantly reducing the number of candidate genes in these examples by using CDG (see Supplementary Material, Section 2 for details). CDG also identifies novel gene candidates for lists of diseases defined by an investigator. Thus, this resource provides a reference for the potential relevance of novel candidate genes to specific disease phenotypes, simplifying the analysis of NGS data.

## Materials and Methods

### CDG Generation

Human Gene Mutation Database is a manually curated database of variants that may be associated with or predisposing to human genetic conditions (16, 17). From the HGMD March 2015 public full version (updated through December 2014), we selected 5,430 HGMD genes classified as high-quality disease-causing or disease-associated mutations (mostly linked to monogenic diseases). We next identified 13,005 protein-coding genes present in the HGC that are not currently reported to be disease-causing in the HGMD database. Briefly, the HGC (15) is a network of all human genes (represented as nodes), where each edge represents the direct biological distance between two human genes. Direct biological distance is defined as the inverse confidence score for binding connectivity provided by STRING (10). The HGC biological distance between any two genes is defined as the weighted sum of direct distances in the shortest path connecting two given genes (calculated using the Dijkstra algorithm), on the network containing most protein-coding human genes.

For each of these 13,005 genes, we calculated their biologically CDG and associated diseases by first retrieving the corresponding connectome for each gene from the HGC database (15, 18). A gene-specific connectome contains, for any given human gene, the set of all other human genes ranked by their biological distance to that specific gene. Then, following the HGC criterion for biological relatedness, we selected only the HGMD known disease-causing genes in the connectome within *p* < 0.01. Additionally, we assigned the corresponding human phenotype ontology codes [HPO (19)] to each gene-phenotype association (Figure S1 in Supplementary Material). A summary of the CDG, diseases, and routes associated with each of the 13,005 genes not currently known to be disease-causing is provided in Table S1 in Supplementary Material.

### Validation

We validated CDG and compared the performance of CDG with FunCoup and HumanNet using a validation set of genes not used during the construction of the original CDG database. As validation set, we used two external datasets (1) a new HGMD dataset, containing 339 disease-causing genes added between January and September 2015 (i.e., not used to construct CDG); and (2) the pathogenic genes from ClinVar not present in HGMD, comprising 84 genes. We calculated the CDG for each of these genes as described above and compared the performance of CDG versus FunCoup and HumanNet in terms of number of predicted genes and how many predicted diseases coincided with the reported disease. As FunCoup and HumanNet do not associate diseases, we retrieved the disease names related to each predicted gene from HGMD. To compare the predicted and expected disease names, we implemented in CDG the following phrase-comparison procedure (1) first, the disease names were compared by exact coincidence. Then (2) using the “starts-with” comparison: if one phrase exactly starts with the other phrase, or *vice versa*. If at this point no matches were found, we used (3) the Levenshtein distance algorithm (20). All comparisons between disease names for the validation dataset were verified manually.

### Data Storage and web Access

To make CDG easily accessible, we created a webserver that allows to consult the CDG database using either genes or diseases as input. If the input gene is known to be disease-causing, the server provides the known associated diseases. And if the gene is unknown to be disease-causing, predicted data is displayed. The server also allows using disease names as input, returning the list of both known and predicted causative genes. The disease names in the CDG database are as reported in HGMD. If the user input is not a HGMD disease name, the procedure to compare disease names described above is used to estimate the closest HGMD disease name.

For the CDG server, MySQL was used to structure and store the multi-dimensional profile of the results of this study, and to process queries to allow efficient access. JSP and servlets were used to parse inputs and generate queries. The web interface is stored on a Rockefeller University Linux-based server in solid state drives. The CDG resource is platform-independent and is freely available to all non-commercial users. The CDG database and server will be periodically updated with new public versions of HGMD, STRING, and HGC.

## Results

### CDG Validation and Comparative Analysis

**Figure 1:**
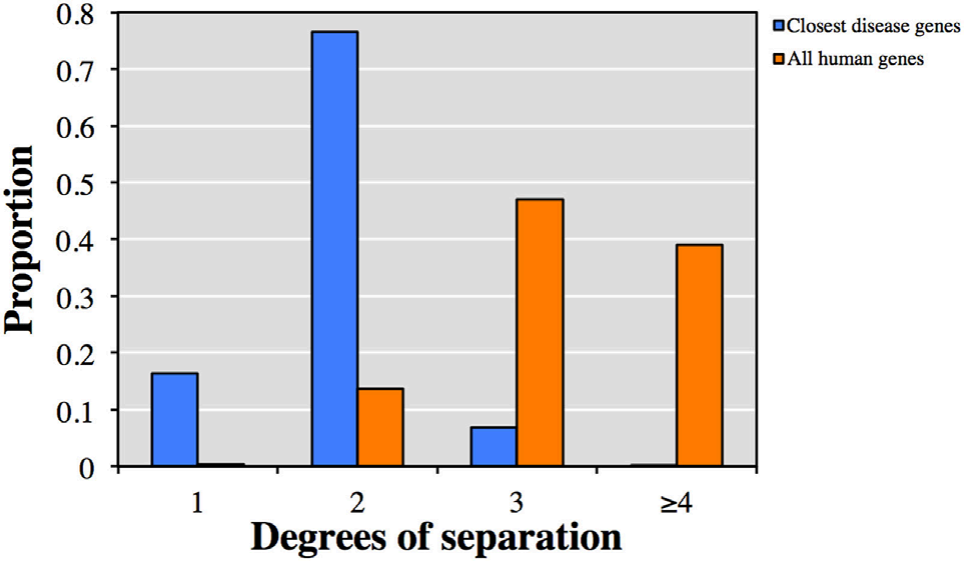
Predicted degrees of separation between (blue) the 13,005 genes from human gene mutation database (HGMD) not known to be disease-causing and their closest predicted HGMD disease-causing genes, and (orange) between all pairs of human genes.

We first explored the relationship between the 13,005 genes not currently described to cause clinical phenotypes with HGMD known disease-causing genes. Each of the 13,005 genes was associated on average with 48 HGMD disease-causing genes and 7 diseases by HGC biological proximity (see Table S1 in Supplementary Material for the top-ranked associations). Notably, 92.9% of the associated disease-causing genes were within one or two degrees of separation from the corresponding query gene (Figure 1). Conversely, only 13.9% of all human gene pairs were within one or two degrees of separation (*p* < 10^−300^, two-tailed equal variance *t*-test).

**Figure 2:**
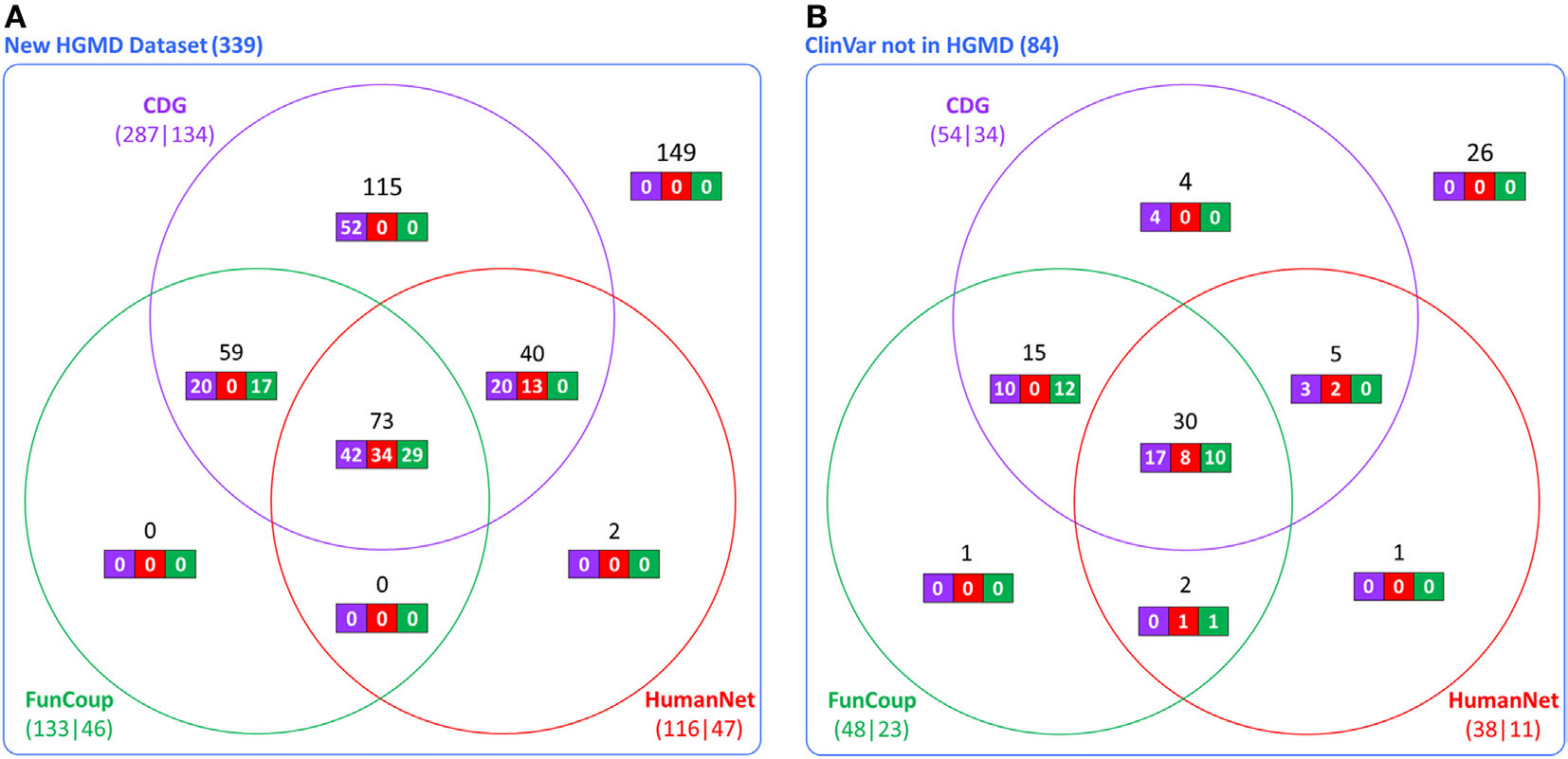
Comparative performance of CDG, FunCoup, and HumanNet using **(A)** 339 new genes in human gene mutation database (HGMD) and **(B)** using 84 genes in ClinVar that are not in HGMD. The numbers below each method show the number of genes with at least one predicted gene (left) and how many were associated with the expected disease (right). Black numbers show the gene distribution across the three servers and white numbers show how many were associated with the expected disease in each server.

The accuracy and utility of these associations was then assessed using new disease-causing genes not known during the construction of the CDG database. Using the first dataset (339 new genes from HGMD), we found that 287 had at least one predicted gene by CDG, compared to 133 using FunCoup and 116 using HumanNet. From these predicted genes, 134 of 287 were associated with the expected disease by CDG, compared to 46 genes of 133 by FunCoup and 47 of 116 by HumanNet (Figure 2A). We repeated the comparison using the second dataset (84 genes from ClinVar not present in HGMD) and observed that CDG similarly outperformed the other two software both in number of genes with at least one predicted disease-causing gene and also in correct association with the expected disease (Figure 2B).

**Figure 3:**
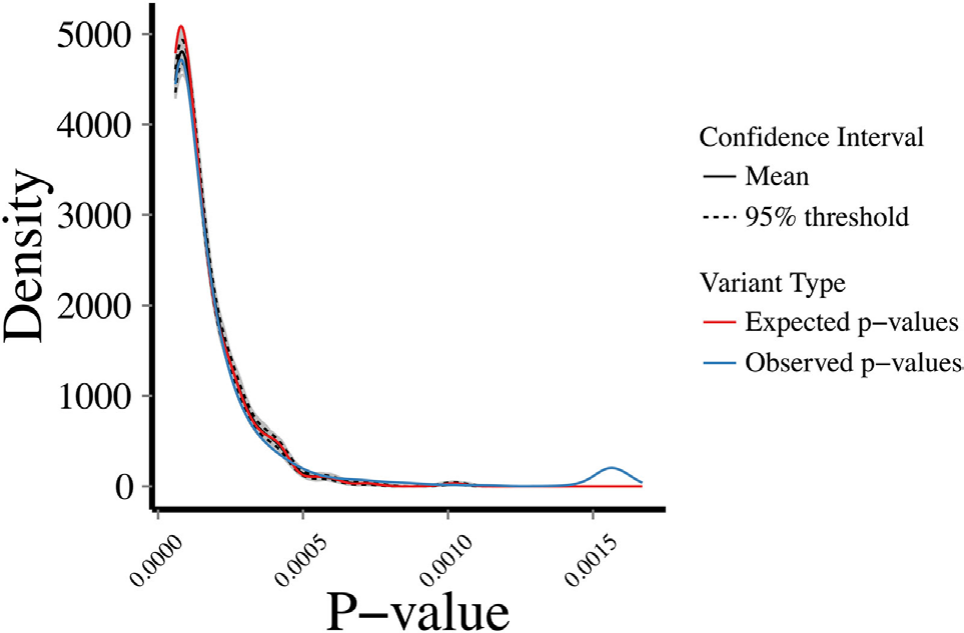
Bootstrapping simulations between a set of (1) expected: *p*-values between 13,005 genes not reported to cause disease and their predicted CDGs; (2) observed: *p*-values between new human gene mutation database genes (i.e., not used to generate the CDGs presented in this study) and their predicted CDGs. Test performed by random sampling using a Gaussian distribution.

To address the robustness of the predictions, we randomly sampled 1,000 sets of 287 genes from the 5,430 known disease-causing genes and estimated their CDGs and associated diseases. CDG identified the expected disease in 86.33% of cases by exact disease name match. Then, we examined the profiles of biological proximity for CDG predictions and known disease-causing genes. Assuming a Gaussian distribution, we performed 10,000 bootstrapping simulations for HGC *p*-values of CDG predictions between the observed 287 new HGMD genes with at least one CDG and the expected set of 13,005 genes not currently known to cause disease. The observed and expected CDG predictions yielded similar *p*-value profiles for biological relatedness between the observed and expected gene sets and their CDG (Figure 3). Therefore, CDG associations are expected to be more robust and relevant for the putative diseases associated with candidate genes than previous methods. Due to the lack of flat files from FunCoup and HumanNet, it was not possible to repeat this analysis with these methods. Thus, we expect that CDG predictions are of significant utility to researchers exploring genes without published phenotypes.

### Examples of CDG Usage

Finally, we demonstrated the utility of CDG in WES data in five patients with various primary immunodeficiencies, modes of inheritance, and known mutated genes that were not in the HGMD public database during CDG generation (extended description and flowchart in Supplementary Material, Section 2). Phenotypes and associated genotypes in these examples include (1) severe autoinflammation, a homozygous mutation in *RNF31* (21); (2) Epidermodysplasia verruciformis, a homozygous mutation in *STK4* (*MST1*) (22); (3) herpes simplex encephalitis, a homozygous mutation in *UNC93B1* (23); (4) common variable immunodeficiency, a heterozygous mutation in *IKZF1* (24); and (5) natural killer cell deficiency, compound heterozygous mutations in *GINS1* (25). The range of initial number of genes per patient was 14,800–18,862. We then applied standard QC (DP > 4, MQ > 40, and QD > 2), minor allele frequency (<1%) (26), and gene-level filtering using GDI (27) and MSC (28), reducing the number of genes in each patient to the range from 18 to 322 candidate genes (numbers mostly dependent on mode of inheritance). Finally, applying the CDG server to the number of genes to investigate reduced this range from 1 to 11, a reduction in candidate genes of 92.1–96.6%, without losing any of the pathogenic genes.

## Conclusion

We provide the first resource by estimating the closest known disease-causing genes and their associated diseases for 13,005 human genes not currently known to be disease-causing. From the comparisons performed, we conclude that CDG predictions capture meaningful candidate disease-causing genes and diseases. We propose to use CDG with lists of genes from NGS studies or similar sources to (1) explore the likelihood of candidate genes being associated with a disease of interest by investigation of its CDGs and associated diseases; (2) rapidly identify known diseases associated with HGMD disease-causing genes; and (3) assign CDGs and associated diseases in variant annotation software. We are also providing an option for users to perform CDG queries based on OMIM (29), although this resource contains less pathogenic mutations compared to HGMD. See Supplementary Material, Section 3 for further details regarding the webserver’s construction.

**Figure 4:**
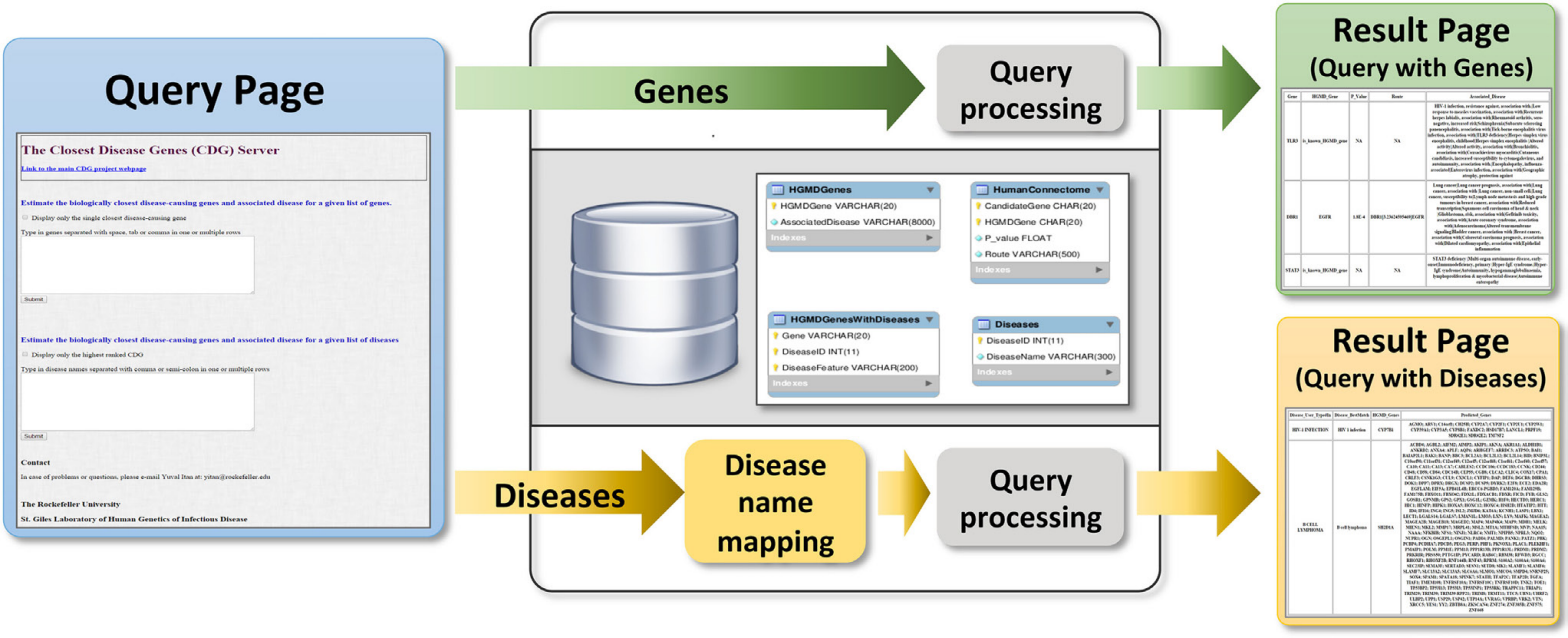
Schematic of the closest disease-causing genes (CDG) server pipeline, where CDG can be estimated by queries of gene or disease lists provided by the user.

Users can submit genes to the webserver (Figure 4) to obtain two outputs (1) all CDGs and associated diseases, including their routes to the input genes (i.e., HGC-predicted genes on the shortest path) and (2) only the most significant CDG for each input gene (by *p*-value). If the input is a known disease-causing gene, the output will be all known associated diseases. Disease names can also be input to obtain known and predicted disease-causing genes for the phenotype concerned. When CDG does not provide desirable results, we propose to rerun it with different diseases that are phenotypically close to the disease that is investigated. We expect that the use of CDG with gene-level filtering methods, such as the gene damage index (27), will facilitate the discovery of new disease-causing genes. The CDG server will be updated when new public versions of HGMD become available, and new features will be added, including filtering by degrees of separation and phenotype matching. The CDG resource and database are available for download from the main CDG webpage: http://lab.rockefeller.edu/casanova/CDG.

## Author Contributions

YI initiated the study. PS, DC, CC-R provided data and expertise. DR, PM, BB, PS, and YI analyzed the data. DR and LS generated the webserver. DR performed the comparison. DR, PM, AA, J-LC, LA, and YI wrote the manuscript. J-LC, LA, and YI supervised the study. All the authors revised and approved the final version of the manuscript.

## Conflict of Interest Statement

The authors declare that the research was conducted in the absence of any commercial or financial relationships that could be construed as a potential conflict of interest.

## Supplementary Material

Table S1 List of all CDGs, their associated HPO IDs and disease names. Also including the p-values and predicted routes between CDGs and candidate genes.
