# Supplementary material for: CDG: An Online Server for Detecting Biologically Closest Disease-Causing Genes and its Application to Primary Immunodeficiency
Source: Front Immunol. 2018 Jun 27;9:1340. doi: 10.3389/fimmu.2018.01340 (PMC6030251; doi:10.3389/fimmu.2018.01340)
Supplement: Supplementary file 2 [file Data_Sheet_1.PDF]

CDG: an online server for detecting biologically closest disease-causing genes and its application to primary immunodeficiency

David Requena[1,‡], Patrick Maffucci[2,3,1,‡], Benedetta Bigio[1], Lei Shang[1], Avinash Abhyankar[4], Bertrand Boisson[1,5,6], Peter D. Stenson[7], David N. Cooper[7], Charlotte Cunningham-Rundles[2,3], Jean-Laurent Casanova[1,5,6,8,9], Laurent Abel[5,6,1], and Yuval Itan[10,11,*]

[1]St. Giles Laboratory of Human Genetics of Infectious Diseases (Rockefeller Branch), The Rockefeller University, New York, NY, USA
[2]Graduate School, Icahn School of Medicine at Mount Sinai, NY, USA
[3]Department of Medicine, Division of Clinical Immunology, Icahn School of Medicine at Mount Sinai, NY, USA
[4]New York Genome Center, New York, NY, USA
[5]Laboratory of Human Genetics of Infectious Diseases (Necker Branch), INSERM U1163, Paris, France
[6]Paris Descartes University, Imagine Institute, Paris, France
[7]Institute of Medical Genetics, School of Medicine, Cardiff University, Cardiff, United Kingdom
[8]Howard Hughes Medical Institute, New York, NY, USA
[9]Pediatric Immunology-Hematology Unit, Necker Hospital for Sick Children, Paris, France.
[10]The Charles Bronfman Institute for Personalized Medicine, Icahn School of Medicine at Mount Sinai, New York, NY, USA
[11]Department of Genetics and Genomics, Icahn School of Medicine at Mount Sinai, New York, NY, USA

‡ Contributed equally.
* To whom correspondence should be addressed.

Correspondence: Yuval Itan, yuval.itan@mssm.edu

Supplementary Material

1. <u>CDG construction:</u>

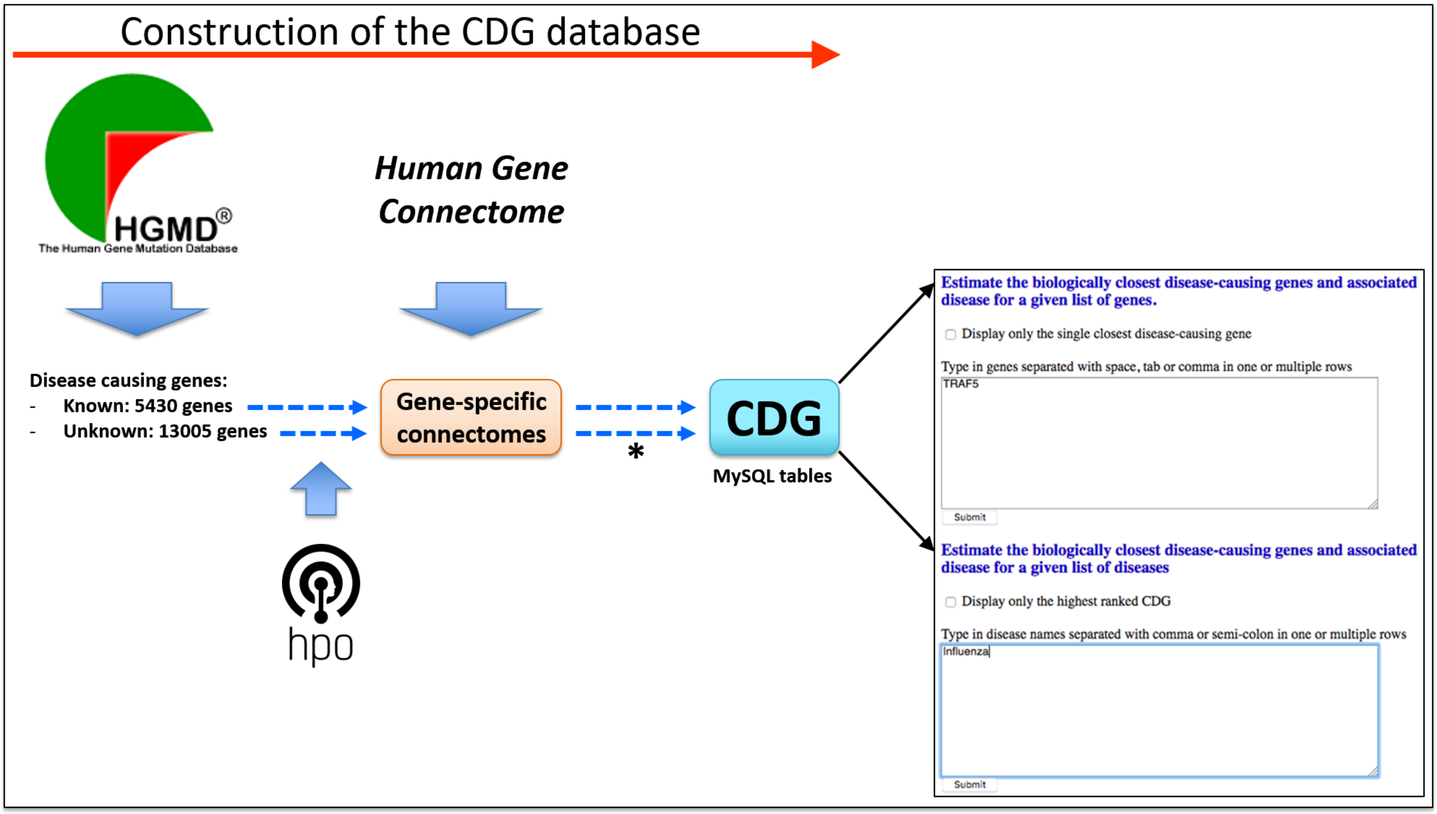

Supplementary Figure 1. Scheme of the CDG construction. The public release of HGMD was used to select genes known to be disease causing and genes that are unknown to be disease causing (UDGs). The corresponding HPO terms were mapped. Their gene-specific connectomes were calculated using the Human Gene Connectome method. For each UDG gene-specific connectome, the related known disease-causing genes with $P < 0.01$ were selected (*), which are defined as the "closest disease-causing genes". Their associated disease terms were associated from the HGMD public release. Finally, MySQL tables were generated for these human protein-coding genes and associated disease terms, to create the CDG database and webpage.

2. <u>Examples using CDG:</u>

To demonstrate the utility of CDG in practical analyses of patient data, we chose exomes of 5 patients suffering from different immune deficiencies with published mutations and different modes of inheritance. The first exome is from a patient in our internal cohort with a recently published disease-causing mutation in *RNF31* (Boisson et al., 2015). The patient's raw exome contained 18,735 genes with at least one observed variant (Supplementary Fig. 2). We filtered this exome with a standard analysis pipeline, including removing low quality variants (DP < 4, MQ < 40, QD < 2), SNPs (minor allele frequency > 1% in the Exome Aggregation Consortium and 1,000 Genomes Project public databases), Gene Damage Index (GDI) High genes (Itan et al., 2015), Mutation Significance Cutoff (MSC) Low variants (Itan et al., 2016), and subsequently focused our analysis on variants in coding regions. Because the patient was born to consanguineous parents, we further restricted our analysis to homozygous variants. This decreased the number of candidate genes to 38, in which the known disease-causing gene (*RNF31*) remained. We submitted this list of 38 candidate genes to the CDG server and obtained 1,234 unique known disease-causing genes in close biological distance to our candidates. We then filtered these known disease-causing genes to include only those within one degree of separation from the matched candidate gene and with associated diseases matching the phenotype of our patient by keywords. When using the keyword "immunodeficiency", the number of candidate genes was reduced to five, whereas using both "immunodeficiency" and "infection" reduced the number to three. In both cases, the known mutation in *RNF31* remained. In this example, use of CDG served to decrease the number of candidate genes in this patient by 86.8% (removed 33 of 38 genes) and 92.1% (removed 35 of 38 genes), depending upon the keywords chosen. Therefore, incorporation of CDG into exome analysis pipelines has the potential to greatly reduce the number of gene and variant candidates that require in-depth computational and experimental analysis.

We repeated the same process for 4 additional exomes of patients: (1) an Epidermodysplasia verruciformis patient with a homozygous mutation in the gene *STK4* (*MST1*). The exome started with 14,800 genes, that reduced down to 18 genes with the standard filtering described above. CDG further narrowed the number of genes to the single published pathogenic gene; (Crequer et al., 2012); (2) a herpes simplex encephalitis patient with a homozygous mutation in the gene *UNC93B1*. Starting with 18,794 genes, the standard pipeline filtered the number of candidates down to 23 genes, which was further reduced by CDG to the single pathogenic gene; (Casrouge et al., 2006); (3) a common variable immunodeficiency patient with a heterozygous mutation in the gene *IKZF1*. Of 18,862 total genes, the standard filtering pipeline yielded 116 candidate genes. CDG narrowed the final number of genes to 9 candidate genes, including the pathogenic gene; and (Kuehn et al., 2016); and (4) a natural killer cell deficiency patient with compound heterozygous mutations in the gene *GINS1*. The patient's exome started with 18,600 genes and was subsequently reduced to 322 genes with standard filtering. CDG narrowed the final number of genes to 11 candidate genes, including the pathogenic gene. (Cottineau et al., 2017). Therefore, incorporation of CDG into exome analysis pipelines has the potential to greatly reduce the number of gene and variant candidates that require in-depth computational and experimental analysis.

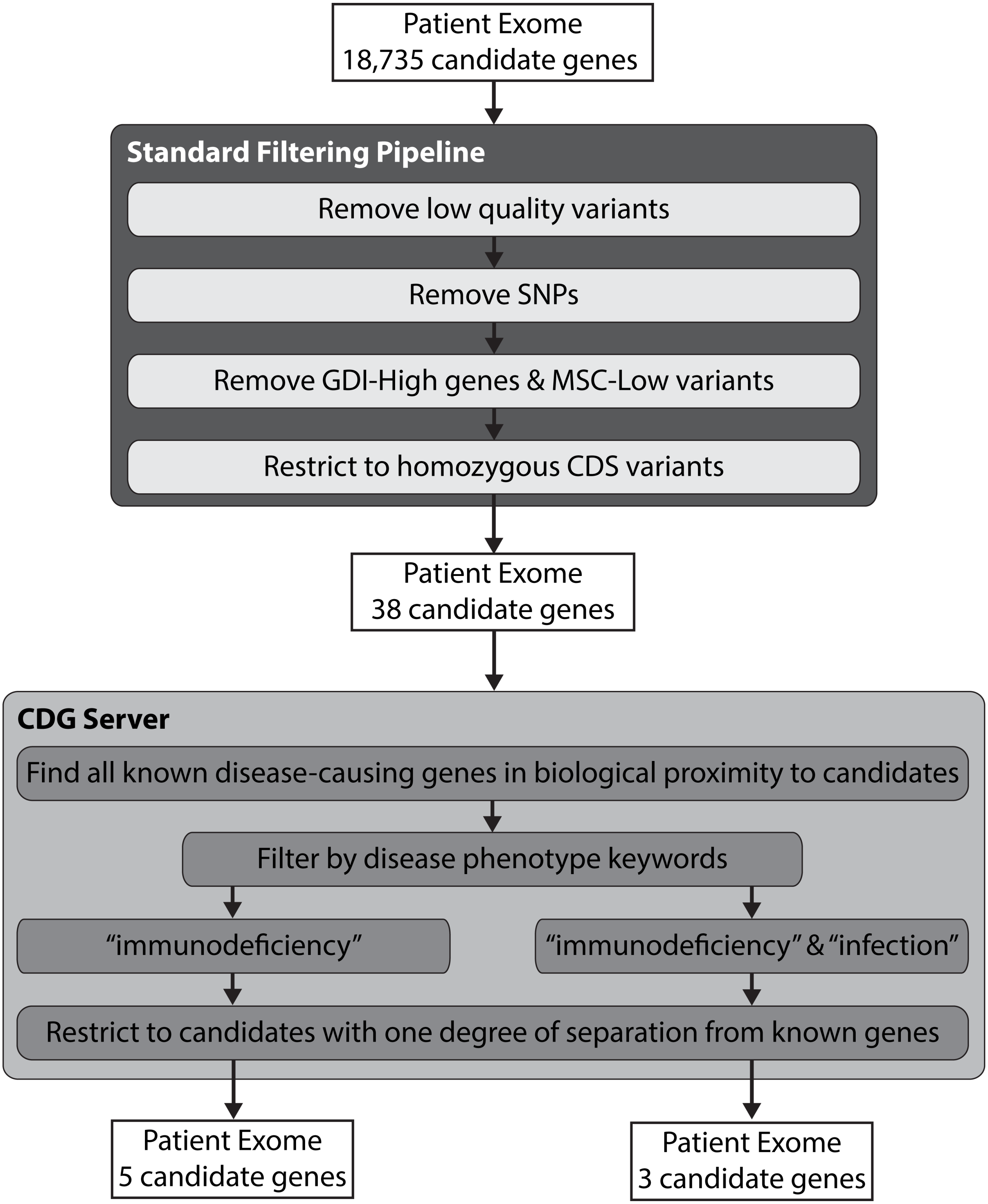

Supplementary Figure 2. Analysis of the exome from a patient with a published disease-causing mutation in RNF31. The exome was first screened with a standard NGS pipeline for candidate variant identification. The 38 candidate genes remaining were processed by the CDG server to identify candidates within close biological distance to HGMD disease-causing genes known to produce similar phenotypes. Depending on the keywords used, use of the CDG server reduced the final number of candidate genes by 86.8% or 92.1%. GDI = Gene Damage Index; MSC = Mutation Significance Cutoff; CDS = coding sequence.

3. OMIM-based CDG:

A CDG alternative version using OMIM as source was prepared. The training dataset included the known gene-disease associations up to December 2015, and the testing dataset all the genes reported later until March 2018. Analogously as done for the HGMD-based CDG, we selected from OMIM only the gene-phenotype associations with a "Confirmed" status and having an approved HGNC symbol. We obtained a preliminary training dataset of 1,736 genes and a testing dataset of 305 genes. Then, just those genes present in the Human Gene Connectome (HGC) were considered, obtaining a final training set of 1,630 genes and a testing set of 280 genes.

As result, 14,933 genes (in the HGC but not in the training dataset) we successfully associated with at least one of the genes of the training dataset, including the corresponding phenotypes. This resulted in 410,775 gene-phenotype predicted associations. Even though all the genes from the testing dataset were associated with at least one phenotype, just 26 (9.29%) matched with the phenotype reported in OMIM.

4. References:

1. Boisson, B., Laplantine, E., Dobbs, K., Cobat, A., Tarantino, N., Hazen, M., et al. (2015). Human HOIP and LUBAC deficiency underlies autoinflammation, immunodeficiency, amylopectinosis, and lymphangiectasia. *J Exp Med* 212(6)**,** 939-951. doi: 10.1084/jem.20141130.
2. Casrouge, A., Zhang, S.Y., Eidenschenk, C., Jouanguy, E., Puel, A., Yang, K., et al. (2006). Herpes simplex virus encephalitis in human UNC-93B deficiency. *Science* 314(5797)**,** 308-312. doi: 10.1126/science.1128346.
3. Cottineau, J., Kottemann, M.C., Lach, F.P., Kang, Y.H., Vely, F., Deenick, E.K., et al. (2017). Inherited GINS1 deficiency underlies growth retardation along with neutropenia and NK cell deficiency. *J Clin Invest* 127(5)**,** 1991-2006. doi: 10.1172/JCI90727.
4. Crequer, A., Picard, C., Patin, E., D'Amico, A., Abhyankar, A., Munzer, M., et al. (2012). Inherited MST1 deficiency underlies susceptibility to EV-HPV infections. *PLoS One* 7(8)**,** e44010. doi: 10.1371/journal.pone.0044010.
5. Itan, Y., Shang, L., Boisson, B., Ciancanelli, M.J., Markle, J.G., Martinez-Barricarte, R., et al. (2016). The mutation significance cutoff: gene-level thresholds for variant predictions. *Nat Methods* 13(2)**,** 109-110. doi: 10.1038/nmeth.3739.
6. Itan, Y., Shang, L., Boisson, B., Patin, E., Bolze, A., Moncada-Velez, M., et al. (2015). The human gene damage index as a gene-level approach to prioritizing exome variants. *Proc Natl Acad Sci U S A* 112(44)**,** 13615-13620. doi: 10.1073/pnas.1518646112.
7. Kuehn, H.S., Boisson, B., Cunningham-Rundles, C., Reichenbach, J., Stray-Pedersen, A., Gelfand, E.W., et al. (2016). Loss of B Cells in Patients with Heterozygous Mutations in IKAROS. *N Engl J Med* 374(11)**,** 1032-1043. doi: 10.1056/NEJMoa1512234.
